# CRISPR/Cas9 system targeting regulatory genes of HIV-1 inhibits viral replication in infected T-cell cultures

**DOI:** 10.1038/s41598-018-26190-1

**Published:** 2018-05-17

**Authors:** Youdiil Ophinni, Mari Inoue, Tomohiro Kotaki, Masanori Kameoka

**Affiliations:** 1 Center for Infectious Diseases, Kobe University Graduate School of Medicine, Hyogo, 650-0017 Japan; 2 Department of International Health, Kobe University Graduate School of Health Sciences, Hyogo, 654-0142 Japan

## Abstract

The CRISPR/Cas9 system provides a novel and promising tool for editing the HIV-1 proviral genome. We designed RNA-guided CRISPR/Cas9 targeting the HIV-1 regulatory genes *tat* and *rev* with guide RNAs (gRNA) selected from each gene based on CRISPR specificity and sequence conservation across six major HIV-1 subtypes. Each gRNA was cloned into lentiCRISPRv2 before co-transfection to create a lentiviral vector and transduction into target cells. CRISPR/Cas9 transduction into 293 T and HeLa cells stably expressing Tat and Rev proteins successfully abolished the expression of each protein relative to that in non-transduced and gRNA-absent vector-transduced cells. Tat functional assays showed significantly reduced HIV-1 promoter-driven luciferase expression after *tat*-CRISPR transduction, while Rev functional assays revealed abolished gp120 expression after *rev*-CRISPR transduction. The target gene was mutated at the Cas9 cleavage site with high frequency and various indel mutations. Conversely, no mutations were detected at off-target sites and Cas9 expression had no effect on cell viability. CRISPR/Cas9 was further tested in persistently and latently HIV-1-infected T-cell lines, in which p24 levels were significantly suppressed even after cytokine reactivation, and multiplexing all six gRNAs further increased efficiency. Thus, the CRISPR/Cas9 system targeting HIV-1 regulatory genes may serve as a favorable means to achieve functional cures.

## Introduction

Human immunodeficiency virus-1 (HIV-1) infection continues to be a major health issue with more than 35 million individuals being infected worldwide^1^. Combination antiretroviral therapy (ART) has been effective at suppressing viremia and restoring primary host CD4+ T cells as well as improving clinical outcomes, thereby transforming a deadly infection into a manageable chronic disease^2^. However, low-level virion production continues to exist due to viral persistence inside cellular (e.g. resting CD4+ T cells, macrophages, and follicular dendritic cells) and anatomical reservoirs (e.g. the central nervous system)^3^. Among these, resting CD4+ T cells have been the most extensively studied and are considered to be the major obstacle to HIV-1 eradication; a small fraction (0.2–16.4/10^6^) of resting CD4+ T cells harbors latent HIV-1 even under ART suppression^4,5^. Current antiviral compounds are incapable of targeting the integrated proviral genome inside these cellular reservoirs and rapid viral rebound ensues after ART cessation^6–8^. Life-long treatment is mandatory, yet long-term medication has also been complicated by issues such as the chronic side effects of ART, adherence difficulties, the emergence of escape mutants, and morbidities in aging populations due to the prolonged life expectancy of people living with HIV-1^9–12^. A single-time intervention to clear viral genomic persistence represents a sensible and promising approach to achieve a HIV-1 functional cure. In latently infected CD4+ T cells, the proviral genome may shift into a dormant state in which inert transcription causes almost no production of viral proteins, and may later be reactivated during T-cell activation to assemble replication-competent viruses^13^. One strategy is to deliberately induce this reactivation, termed the “shock and kill” approach, using latency-reversing agents such as inhibitors of histone deacetylase (HDACi), and this method has shown the potent induction of T-cell activation followed by viral mRNA increases^14^; however, *ex vivo* assays suggested that the outgrowth of HIV-1 from latent reservoirs is insufficient and cytotoxic T-cell responses are not sufficiently strong to eliminate reactivated infected cells, resulting in a minimal impact on the overall reservoir size^15,16^. These findings indicate that strategies for a HIV-1 cure need to involve the direct disruption of the proviral genome from the cellular reservoir, which may be achieved with site-specific genome editing.

Over the last two decades, advances have been achieved in genome editing technology through the innovation of site-directed engineered nucleases, such as zinc finger nuclease (ZFN) and transcription activator-like effector nuclease (TALEN), which uses the DNA-protein recognition principle to direct FokI nuclease towards essentially any sequence within the genome and digest it^17,18^. However, difficulties associated with design, synthesis, and protein validation for a specific gene locus of interest have restricted the feasibility of these methods^19^. A key breakthrough was made when a bacterial immune system-related RNA molecule, called the clustered regularly interspaced short palindromic repeats (CRISPR), was found to be able to guide CRISPR-associated 9 (Cas9) nuclease towards DNA sequences matching those of the guide RNA (gRNA). This gRNA is easily programmable and the simple transduction of the designed gRNA with a Cas9 expression cassette may introduce double-strand breaks (DSB) inside DNA in a highly specific and efficient manner^20^. CRISPR also has the advantage over ZFN and TALEN of being a smaller size and, thus, is easier to package into lentiviral constructs, has a lower risk of off-target cleavage, is easier to create, less costly, and has demonstrated higher efficiency^19,21^. The CRISPR/Cas9 system has achieved successful outcomes in many mammalian culture cells, including human T-cell lines^22^ and pluripotent cell lines, and has been tried-and-tested in a broad range of *in vitro* and *in vivo* studies on human genetic and infectious diseases^23,24^, including HIV-1^25^.

The successful late transcription of HIV-1 following viral activation is highly dependent on the early expression of the regulatory proteins Tat and Rev. The elongation of nascent viral mRNA from the integrated provirus is initiated by Tat, while the nuclear export of unspliced transcripts is regulated by Rev^26,27^. In HIV-1-infected activated T cells, the combination of Tat and Rev provide a very high level of viral gene expression, while the same proteins in resting T cells are important for maintaining the provirus in a latent state^28^. *tat* and *rev* are considered to be some of the most functionally conserved genes of HIV-1, with some genomic domains inside sharing the same homology across wide HIV-1 subtypes and even to HIV-2 and simian immunodeficiency virus (SIV)^28,29^. Many RNA-based^30–35^ and protein-based^33,36–38^ anti-HIV-1 moieties targeting these proteins or their exons have been successfully shown to reduce viral replication in T cells to a varying degree with methods including, but not being limited to Tat/Rev short hairpin RNA (shRNA), antisense RNA, a trans-activation response/Rev response element (TAR/RRE) decoy, mutant molecules, and *tat/rev*-targeting ribozymes. CRISPR/Cas9 provides a promising new method to target the regulatory genes of HIV-1. In the present study, we constructed a lentiviral vector-based CRISPR/Cas9 system harboring gRNAs with the ability to recognize specific DNA sequences inside the coding sequences of Tat and Rev. We demonstrated that the CRISPR/Cas9 system abolished Tat and Rev protein expression and their respective regulatory functions in stable Tat- and Rev-expressing 293 T cells. Target-site sequencing confirmed that the Cas9-associated mutation occurred inside the *tat* and *rev* exons, while no off-target mutations were detected in sequences similar to the designed gRNAs inside the human genome. We ultimately found that CRISPR transduction successfully diminished viral capsid production in persistently and latently infected CD4+ T-cell lines. These results support the potential use of CRISPR to specifically target HIV-1 regulatory genes and suppress viral replication.

## Results

### CRISPR/Cas9 abolished the expression and function of Tat and Rev proteins

**Figure 1:**
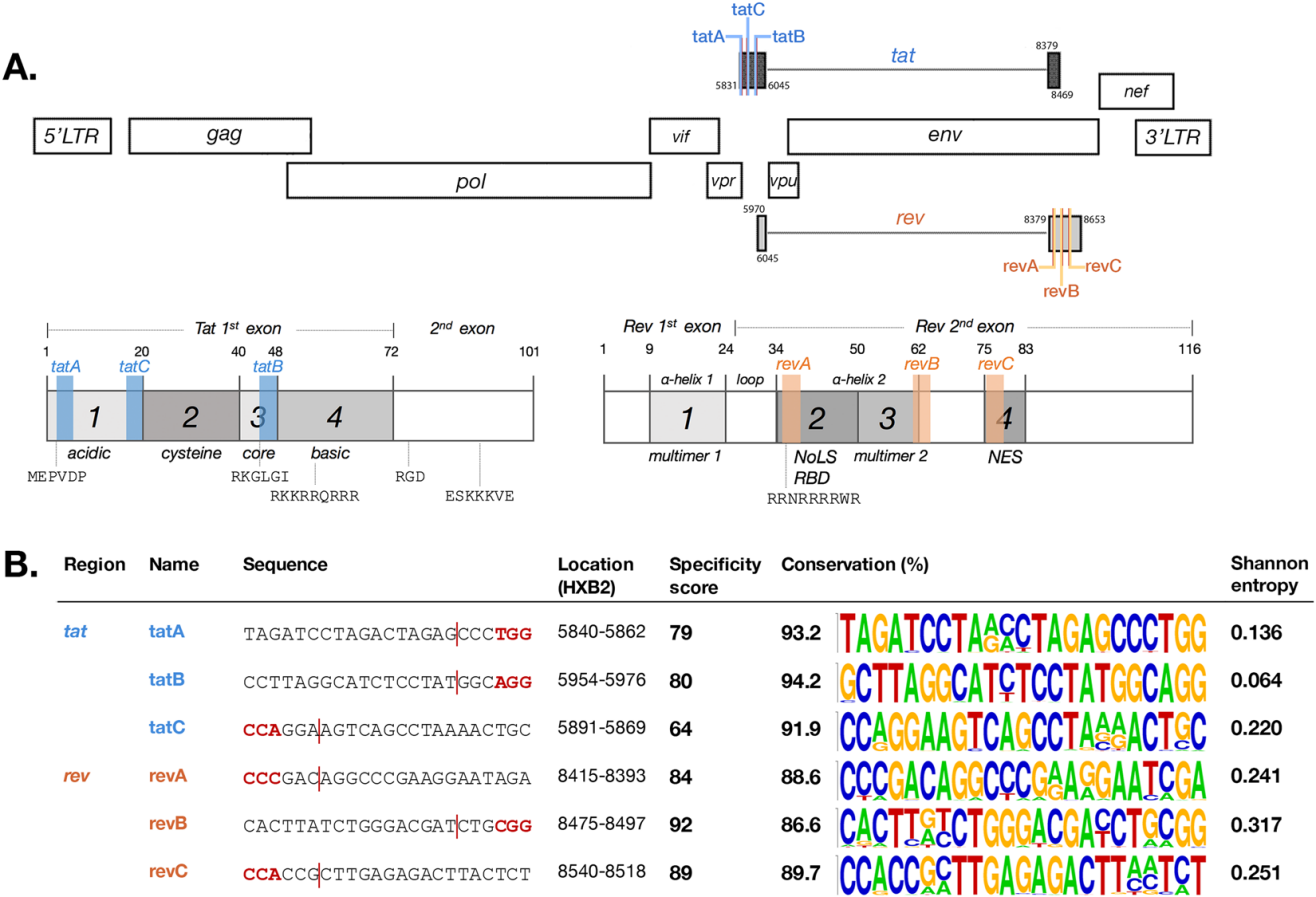
CRISPR gRNAs targeting HIV-1 regulatory genes designed and tested in this study. (**A**) Schematic representation of the HIV-1 proviral genome with the approximate location of each gRNA. Three target sequences were selected from each *tat* and *rev* region: *tatA*, *tatB*, *tatC*, *revA*, *revB*, and *revC*. The specific domains targeted in each gene are shown. (**B**) Sequences of gRNAs with the PAM region highlighted in red and the Cas9 cleavage site 3-nt upstream of PAM denoted by a red line, and respective coordinates in HXB2. CRISPR specificity scores were calculated by algorithms available in http://crispr.mit.edu and the conservation of each sequence was performed across 97 HIV-1 strains of six subtypes (A, B, C, D, 01AE, and 02AG) as curated from the Los Alamos National Lab (LANL) HIV Sequence Compendium. Conservation percentages and nucleotide variabilities as measured by Shannon entropy are shown.

We designed six gRNAs with three constructs targeting each *tat* and *rev* gene (Fig. 1A). All gRNAs contained a 20-bp sequence from targeted genes followed by the 3-bp CRISPR recognition site, called the protospacer adjacent motif (PAM, *NGG*). Three *tat*-targeting gRNAs were taken from the first exon of *tat* with *tatA* targeting the N-terminal acidic domain, *tatB* targeting the short core domain hitting the highly conserved RKGLGI motif, and *tatC* targeting the end of the acidic domain to the start of cysteine residues. Three *rev*-targeting gRNAs were taken from the second exon of *rev* with *revA* targeting the arginine-rich motif in the nuclear localization signal and the RNA-binding domain, *revB* targeting the second multimerization domain, which is crucial for forming the alpha-helical secondary structure of the Rev protein, and *revC* targeting the leucine-rich nuclear export signal effector domain. CRISPR specificity scores were counted based on on-target activity minus the weighted-sum of off-target probability as calculated by software in http://crispr.mit.edu. Selected sequences have relatively high hit scores in this algorithm. The designed 23-bp gRNAs were aligned to 97 HIV-1 strains from subtypes A, B, C, D, CRF01AE, and CRF02AG as curated from the Los Alamos National Laboratory (LANL) database and showed relatively high sequence homology at each site (Fig. 1B).

**Figure 2:**
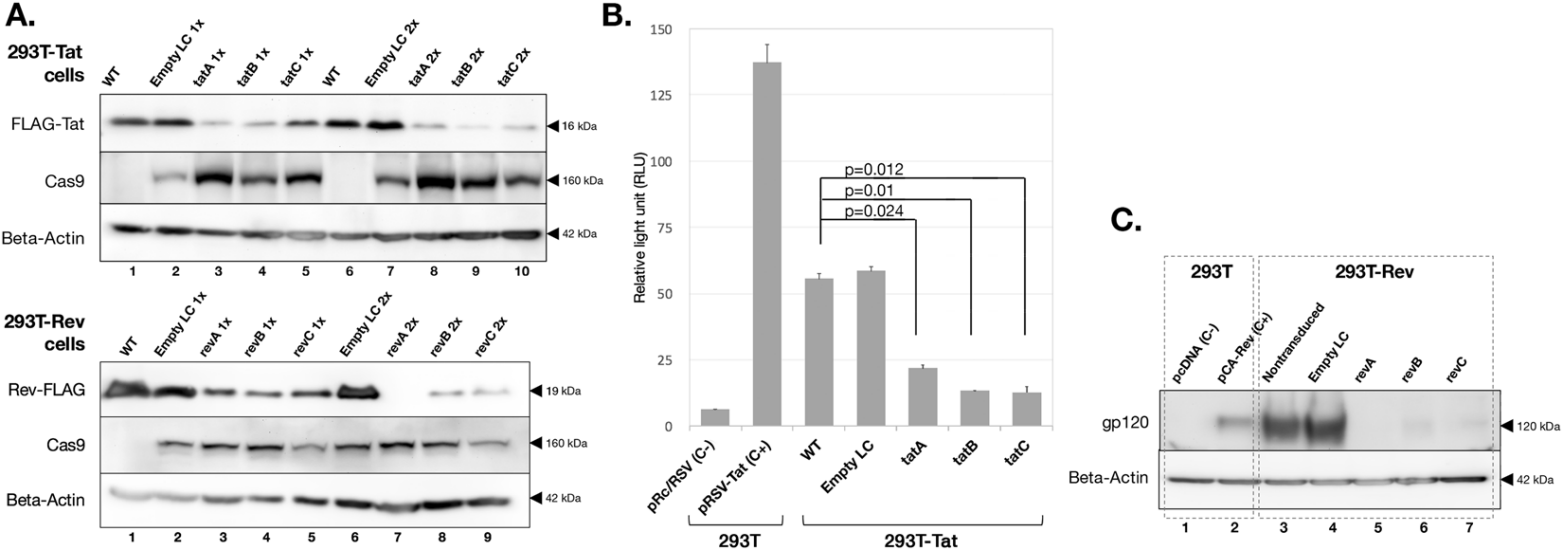
CRISPR/Cas9 abolished Tat and Rev protein expression and function in stable Tat- and Rev-expressing transformant 293 T cells. (**A**) Western blot analysis for FLAG-bound Tat and Rev protein expression. Transformant 293 T cells were transduced with a CRISPR/Cas9-bearing lentiviral vector for 3 days before being lysed and immunostained with an anti-FLAG antibody. The transduction of lentiCRISPR without gRNA (empty LC) and with *tat*/*rev*-targeting gRNAs was performed once or twice, and protein expression was compared with non-transduced cells (WT). The Cas9 protein was detected by an anti-Cas9 antibody and expression was shown for each cell group. Beta-actin expression was used as the loading control. Assays were performed at least twice for the expression of each protein and the most representative image was selected. Original unedited blots are shown in the supplementary dataset and images were not joined from different parts of the gel. (**B**) A Tat functional analysis of 293T-Tat cells. WT and CRISPR-transduced cells were transfected with pLTR-Luc for 3 days and cell lysates were obtained to measure luciferase activity. The transfections of pRc/RSV and the Tat expression vector pRSV-Tat into original 293 T cells were used as negative and positive controls, respectively. A one-way ANOVA applied for five groups (WT, empty LC, *tatA*, *tatB*, and *tatC*) gave a *p* value < 0.001. Pairwise comparisons between WT and CRISPR-transduced cells are shown. Experiments were performed three times and average values ±SE are shown. (**C**) A Rev functional analysis of 293T-Rev cells. WT and CRISPR-transduced cells were transfected with the gp140-expressing plasmid for 3 days and the cell lysate was immunostained with an anti-gp120 antibody. The transfections of pcDNA3.1/Zeo(+) and the Rev expression plasmid pCA-Rev into original 293 T cells were used as negative and positive controls, respectively. Assays were performed twice and the most representative image was selected. Original unedited blots are shown in the supplementary dataset and images were not joined from different parts of the gel.

In order to analyze the effects of the designed CRISPR/Cas9 construct to HIV-1 regulatory protein expression, we first developed stable, FLAG-bound Tat- and Rev-expressing HEK 293 T cell lines by infecting cells with a lentiviral vector bearing FLAG-tagged HIV-1 NL4-3 *tat* and *rev*, mimicking actual HIV-1 infection. These transformant cells constantly produced FLAG-bound Tat and Rev proteins at high amounts, as confirmed by an anti-FLAG immunoblot assay. Vesicular stomatitis virus glycoprotein (VSV-G) pseudotyped lentiviral vectors expressing *S. pyogenes* Cas9 nuclease bearing the designed gRNAs were then transduced into 293T-Tat and 293T-Rev cells, either once or twice with a multiplicity of infection (MOI) of 10. MOI of 10 was selected because it was previously shown to be the most optimal for expressing transgenes into target T cells with minimal cytotoxicity^39^. In addition, the designed gRNAs were not able to target the HIV-1-based lentiviral vectors used for transduction. The lentiviral backbone lentiCRISPRv2 (LC) harbored long terminal repeat (*LTR*) and *RRE* genes, but did not regulate the expression of U6 transcription-based gRNA or Cas9. Medium was maintained for five days for each transduction and the cell lysate was then assayed using an immunoblot analysis. Western blot results showed that *tat*- and *rev*-CRISPR abolished Tat and Rev expression relative to that in non-transduced cells (WT). A second round of transduction further reduced protein expression (Fig. 2A). A marked reduction in FLAG expression was not observed in cells transduced either once or twice with empty LC, which bore the Cas9 construct without gRNA. On the other hand, the Cas9 protein was expressed in all transduced cells, including empty LC-transduced cells, although expression levels varied to some extent. The same treatment of stable FLAG-Tat- and Rev-expressing HeLa cells provided similar results (see Supplementary Fig. S1). Third-round transduction did not result in further marked reductions in Tat-FLAG protein expression or HIV-1 LTR-driven luciferase transcriptional activity (data not shown); however, two-round transduction resulted in a low level of protein expression.

The high-level activation of HIV-1 RNA polymerase depends on the binding of Tat-associated kinase and the TAR element inside HIV-1 3′LTR. We used this principle to perform a functional assay on the Tat protein. Previously established 293T-Tat transformant cells, either non-transduced or transduced with empty LC or *tat-*CRISPR, were transfected with pLTR-Luc for 3 days before being lysed to measure luciferase activity. The plasmid pLTR-Luc contained HIV-1 3′LTR, which drives luciferase expression upon transactivation by the Tat protein onto TAR. The luciferase assay showed that *tat*-CRISPR significantly suppressed HIV-1 LTR-driven expression in 293T-Tat cells by 61%, 76%, and 77% for *tatA*, *tatB*, and *tatC* (P = 0.024, P = 0.01, and P = 0.012), respectively (Fig. 2B). A similar assay using HeLa-Tat cells showed even stronger suppression: 97%, 97%, and 94% (P = 0.007, P = 0.007, and P = 0.008, see Supplementary Fig. S1), respectively, presumably due to lower transduction efficiency during the establishment of transformant HeLa cells and, thus, weaker initial Tat expression. Conversely, a Rev functional assay was performed by comparing gp120 envelope expression because the nuclear export of incompletely spliced *env* mRNA depends on the binding of Rev and the RRE RNA stem loop inside *env*. Control and CRISPR-transduced 293T-Rev cells were transfected with the gp140-expressing plasmid for 3 days before being lysed and immunostained with an anti-gp120 antibody. Western blotting showed the abolished expression of gp120 in 293 T cells treated with *rev*-CRISPR constructs relative to that in the control and cells transduced with empty LC (Fig. 2C), which was, thus, attributed to failed *env* mRNA export due to Rev protein dysfunction. Overall, the results of functional assays on the Tat and Rev proteins appeared to be in agreement with FLAG-Tat and Rev-FLAG expression levels.

### High frequencies of various types of mutations were found precisely at the site of Cas9 DSB

**Figure 3:**
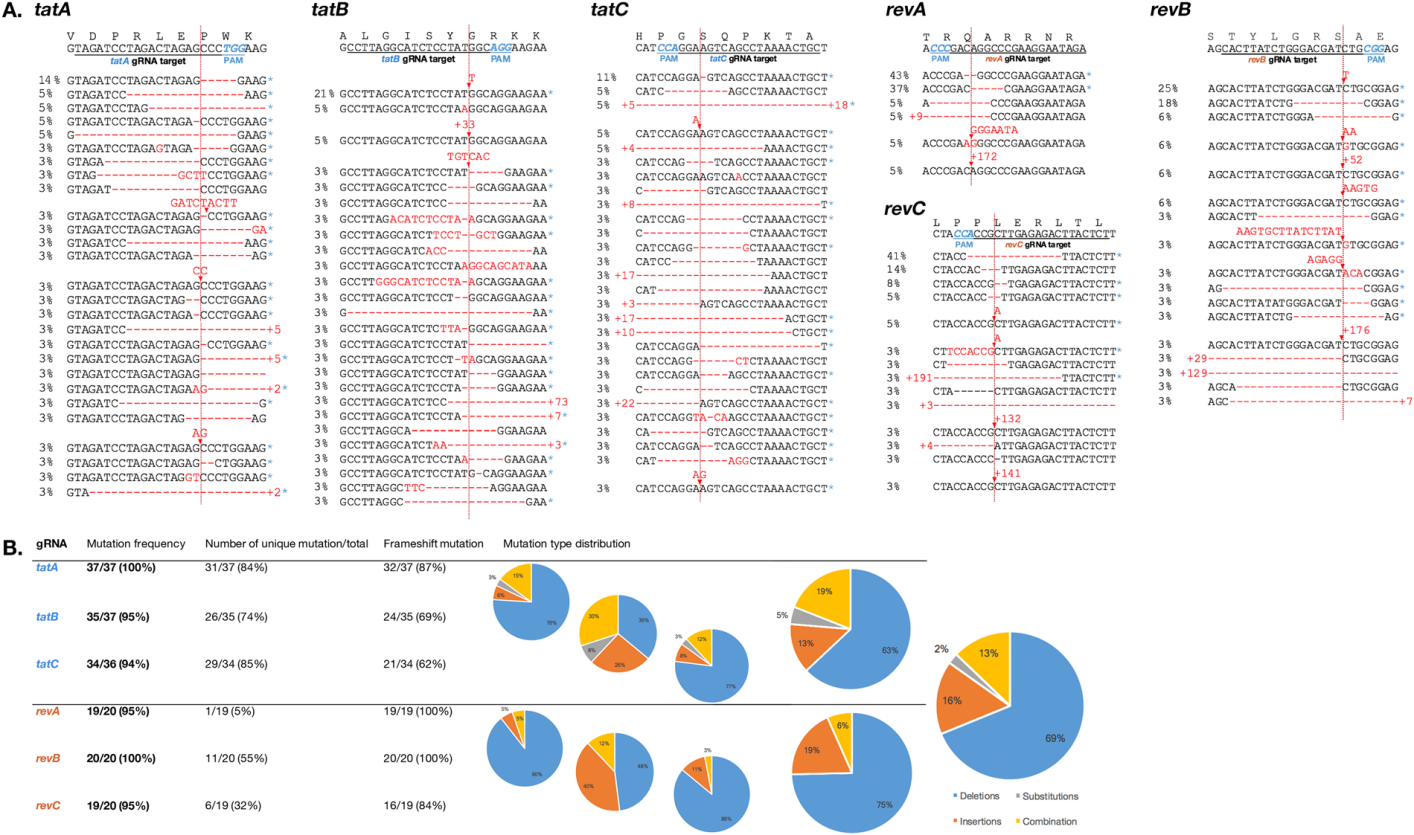
Sequencing analysis of the on-target site for each CRISPR gRNA. (**A**) On-target sites were amplified from the extracted DNA of CRISPR-transduced 293 T cells in order to create at least 20 plasmid clones for each gRNA construct, and amplicons were Sanger sequenced. Wild-type NL4-3 sequences are shown topmost with the PAM site (*NGG*) indicated in blue. Mutations are indicated in red and frameshift mutations are denoted with a blue asterisk (*). Cas9 cleavage sites are indicated in red vertical lines. Percentage numbers at the left side of each sequence denote the frequency of the corresponding mutation pattern found within all sequenced samples for each gRNA. (**B**) Mutation frequency and distribution of mutation types for each gRNA, cumulative for *tat*-CRISPR and *rev*-CRISPR, and overall CRISPR-transduced samples.

In order to directly confirm the nuclease activity of CRISPR/Cas9 inside the HIV-1 proviral genome, we compared the on-target base sequences of CRISPR-transduced cells with a reference. The target regions of all six gRNA from *tat*- and *rev*-CRISPR were amplified from the extracted DNA of CRISPR-transduced 293 T cells to create plasmids, with at least 20 clones for each gRNA construct, and amplicons were subjected to Sanger sequencing (Fig. 3A). Alignment with the NL4-3 wild-type sequence showed that 106/110 clones (96.4%) for *tat* and 58/60 (96.7%) clones for *rev* had mutations. A sequence analysis showed 86 and 16 types of unique patterns of mutations in *tat* and *rev-*CRISPR amplicons, respectively, with 77/106 (72.6%) for *tat* and 56/58 (96.6%) for *rev* being frameshift mutations. Conversely, no mutations were found in the same number of plasmid clones extracted from wild-type cells and empty LC-transduced cellular DNA. Mutations in CRISPR-transduced cells were mostly deletions (63% and 75% for *tat* and *rev*, respectively) followed by insertions (13%, 19%), indel combinations (19%, 6%), and substitutions (5%, 0%), as charted in Fig. 3B. These mutation types are typically observed in the non-homologous end joining (NHEJ)-dependent repair of DNA DSBs, often expected as a result of genome editing without any extrachromosomal template, and the high frequency of deletions was consistent with the patterns observed in CRISPR/Cas9-induced mutations^40^. The indel sites of all mutated samples were located 3-nt upstream of the PAM recognition site (red vertical line in Fig. 3A), which was the Cas9 nuclease cleavage site. Collectively, these results strongly indicated that NHEJ repair-induced mutations found inside CRISPR-transduced cells were caused by CRISPR/Cas9 enzymatic activity.

### CRISPR/Cas9 transduction showed no changes in cell viability and no detectable off-target mutations on the human genome

**Figure 4:**
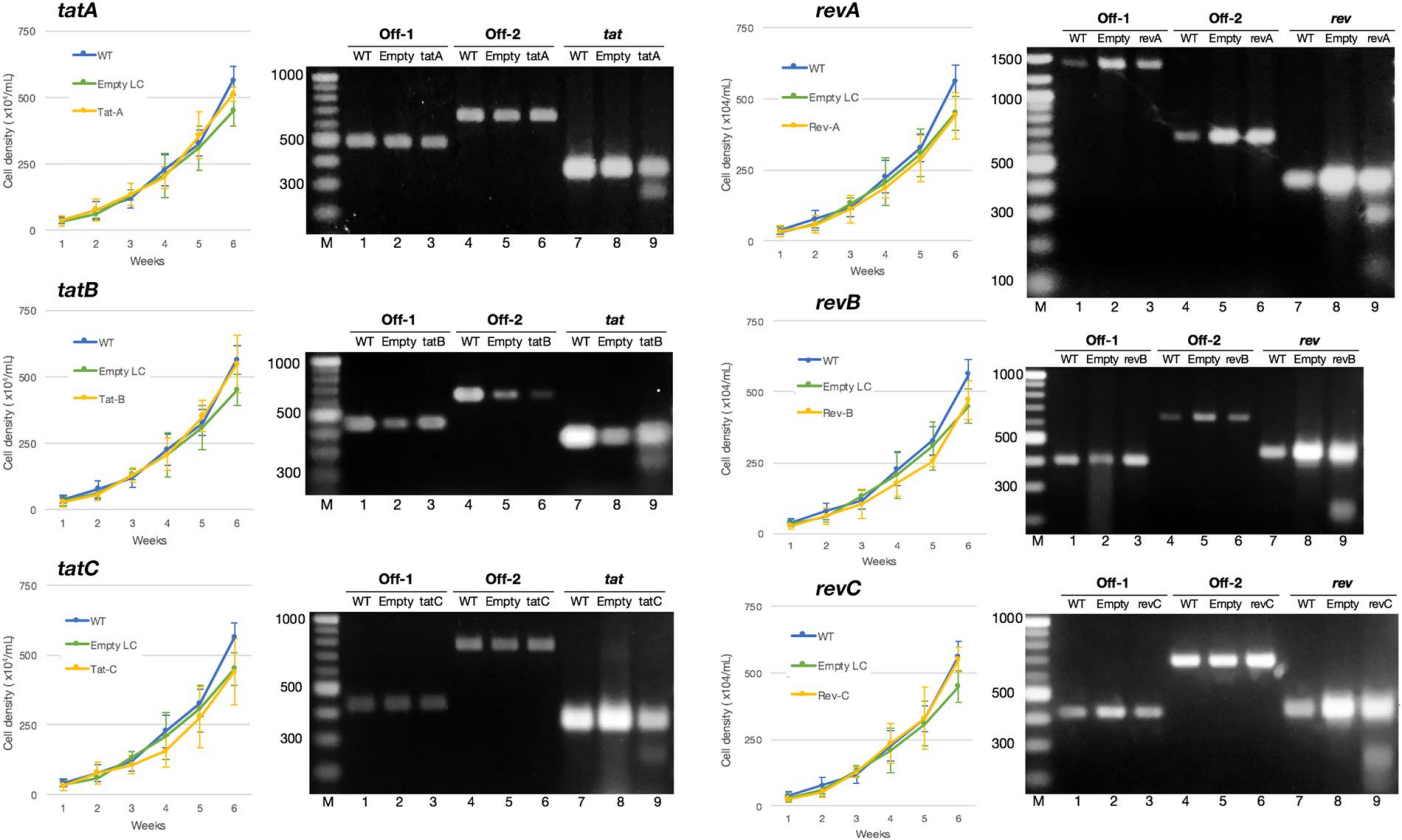
CRISPR/Cas9 transduction showed no changes in cell viability or detectable off-target activity in the human genome. (***Left***) Wild-type, empty lentiCRISPR-transduced, and CRISPR-transduced (blue, green, and yellow lines, respectively) L-2 CD4^+^ T cells were plated at the same density, cell growth was monitored for up to 6 weeks, and viable cells were counted using trypan blue exclusion. Data for each gRNA construct are shown. Experiments were performed three times with average values ±SE as shown. (***Right***) Surveyor mutation detection assays of two off-target sites in the human genome with the highest predicted probability per gRNA construct and corresponding on-target sites as the positive control. A detailed list of off-target sites is shown in Supplementary Table 1. Assays were performed twice for each gRNA and the most representative image was selected. Images were not joined from different parts of the gel.

The recognition mechanism of CRISPR/Cas9 is known to tolerate single and double sequence mismatches between gRNA and target DNA, depending on the position along the gRNA-DNA interface, but particularly in nucleotides distal to the PAM region, and promotes undesired off-target mutations that may introduce genotoxicity and impair cell viability^41^. A cell viability assay was conducted in order to examine the effects of our CRISPR/Cas9 construct on T cells. Wild-type, empty LC-transduced, and CRISPR-transduced L-2 cells were plated at the same density and subsequent cell growth was monitored for up to 6 weeks; no marked differences were found in cell counts using trypan blue exclusion (Fig. 4, left panel). We also investigated the probability of off-target digestion for each gRNA inside the cellular genome by taking into account the total number of nucleotide mismatches, mean pairwise distance between mismatches, and base distance to the PAM recognition site as calculated by the algorithm in http://crispr.mit.edu. Two off-target sites per gRNA with the highest predicted probability of mutations (Supplementary Table 1) were amplified from cellular DNA extracted from CRISPR-transduced cells. Amplicons were then hybridized to form homo- and heteroduplexes and screened using a Surveyor mutation detection assay, in which the presence of any mutation will show as Surveyor nuclease cleavage products with higher mobility inside an agarose gel than the original amplicon. We herein demonstrated that the gel electrophoresis of off-target amplicons for each gRNA showed no apparent cleavage product, while *tat* and *rev* on-target control amplicons both showed the presence of mutations (Fig. 4, right panel).

### CRISPR/Cas9 suppressed HIV-1 replication in persistently and latently infected human CD4+ T-cell lines

L-2 cells are a human CD4^+^ T-cell line harboring the LAV strain of HIV-1 and persistently release a defective p24 capsid protein into the supernatant^42^. gRNA/CRISPR constructs were transduced into these cells for 5 days with MOI 10, the culture was changed to fresh medium, and p24 production in the supernatant was measured to compare HIV-1 replication. Since combination therapy is considered to increase inhibition potency, multiple gRNA transductions with a combination cocktail of three *tat* and *rev* gRNA-bearing lentiviral vectors (*tatABC* and *revABC*) and six gRNAs (*tatrevABC*) were also performed. Lentivirus concentrations and MOI were maintained in all multiplex gRNA treatment groups, i.e. a 1/3 titer or MOI 3.33 for each gRNA/CRISPR vector for *tatABC* and *revABC*, and a 1/6 titer or MOI 1.67 for each vector for *tatrevABC*. ELISA measurements clearly showed that p24 production was significantly lower in all single CRISPR-transduced cells than in WT (P = 0.001, 0.001, 0.001, 0.003, 0.001, and 0.003 for *tatA*, *tatB*, *tatC*, *revA*, *revB*, and *revC*, respectively), with the highest efficacy of a 48-fold reduction being observed in *revB* and no significant reduction being noted between empty LC-transduced cells and WT (P = 0.932). Multiple gRNA treatments resulted in similar or even lower p24 levels than those of single gRNAs (P = 0.003, 0.002, and 0.002 for *tatABC*, *revABC*, and *tatrevABC*, respectively, versus WT).

Latent HIV-1 infection in resting CD4^+^ T cells is the primary cause of the barrier to a functional cure. In order to establish whether our CRISPR constructs reached this cell population, we used J-Lat 10.6, a Jurkat CD4^+^ T-cell clone latently infected with the full-length R7 strain of HIV-1 minus *env* and *nef*^13^. HIV-1 transcription was silent in these cells due to a modified chromatin structure that prevents the access of the cellular transcriptional machinery to the HIV-1 LTR promoter. The treatment of cells with cytokines, such as TNFα, will induce chromatin remodeling, providing access to the HIV-1 promoter, its activation, and the initiation of viral protein production. J-Lat cells were transduced with single and multiple gRNA/CRISPR for five days similar to previous treatments on L-2 cells, the culture was changed to fresh medium, stimulated with 20 ng/mL TNFα, and p24 levels in the supernatant were measured 24 hours after induction (Fig. 4B). ELISA results showed that p24 production after reactivation was significantly lower in CRISPR-transduced J-Lat cells than in WT (P = 0.003, 0.003, 0.008, 0.01, 0.005, and 0.024 for *tatA*, *tatB*, *tatC*, *revA*, *revB*, and *revC*, respectively), with the highest efficacy of a 53-fold reduction being observed in *tatA* and no significant difference being noted between WT and empty LC-transduced cells (P = 0.132). Multiplex gRNA treatments showed similarly low or even lower p24 levels (P = 0.003, 0.003, and 0.003 for *tatABC*, *revABC*, and *tatrevABC*, respectively, versus WT). These results demonstrated that the lentiviral delivery of CRISPR/Cas9 successfully reached the isolated proviral genome, cleaved regulatory genes, and significantly inhibited viral replication, even after latency reversal.

**Figure 5:**
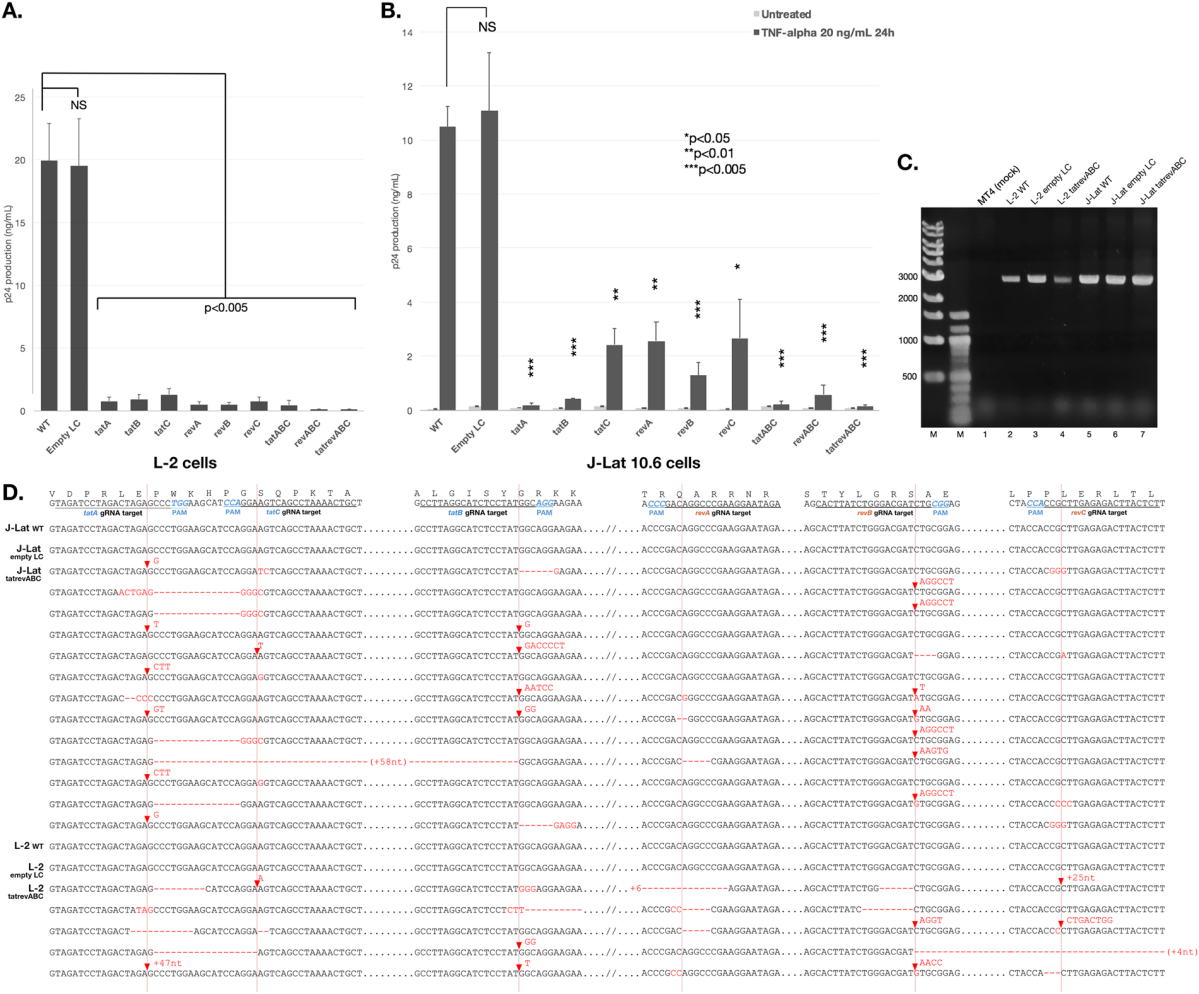
Efficiency of the CRISPR/Cas9 construct in persistent and latent HIV-1 infection. (**A**) Persistently HIV-1-infected CD4^+^ T cells (L-2) were CRISPR-transduced with or without *tat/rev-*targeting gRNAs, either a single or multiple of three (*tatABC* and *revABC*) or six (*tatrevABC*). Transduction was performed for 5 days with MOI 10, the culture was changed to fresh medium, and p24 levels in the supernatant were measured 3 days later. A one-way ANOVA applied to all groups gave a *p* value < 0.001 and pairwise comparisons between WT and all other groups are shown. Assays were performed three times and average values (±SE) are shown. (**B**) Latently HIV-1-infected Jurkat T cells (J-Lat 10.6) were CRISPR-transduced with or without gRNAs similar to the treatment of L-2 cells. Transduction was performed for 5 days with MOI 10, the culture was changed to fresh medium, treated with or without TNFα 20 ng/mL, and p24 levels in the supernatant were measured 24 hours later. A one-way ANOVA applied to all groups gave a *p* value < 0.001 and pairwise comparisons between WT and all other groups are shown. Assays were performed three times and average values ±SE are shown. (**C**) Multiplexing gRNA failed to show excision between *tat* and *rev*. PCR amplification spanning 2876 bp between upstream of the *tat* first exon to the end of the *rev* second exon was conducted using DNA extracted from *tatrevABC*-transduced L-2 and J-Lat cells. Assays were performed three times and representative gel image are shown. The image was not joined from different parts of the gel. (**D**) Amplicons were Sanger sequenced and on-target gRNA sites from WT, empty LC, and several *tatrevABC*-transduced J-Lat and L-2 clones are shown. Wild-type NL4-3 sequences are shown topmost with the PAM site (*NGG*) indicated in blue. Mutations are indicated in red and Cas9 cleavage sites are indicated in red vertical lines.

Multiplexing gRNA/CRISPR constructs opens up the possibility of genomic excision occurring between two gRNA sites. In order to confirm this, PCR amplification spanning 2876 bp from upstream of the *tat* first exon to the end of the *rev* second exon was performed using DNA extracted from *tatrevABC*-transduced L-2 and J-Lat cells. DNA from WT and empty-LC transduced L-2 and J-Lat cells, as well as human T cell leukemia MT-4 cells as a mock sample, were amplified (Fig. 5C). Since all samples, excluding the mock, showed similar ~3000 bp amplicons, it was concluded that no excision was found between the *tat* first exon to *rev* second exon. The sequencing of amplicons further showed that mutations occurred at gRNA sites, albeit without any large excision between *tat* and *rev*. However, two samples showed smaller scale excisions: a 114-bp excision between *tatA* and *tatC* sites and a 54-bp excision between *revB* and *revC* in J-Lat and L-2 cells, respectively. Ultimately, concurrent mutations in multiple gRNA sites were evident in *tatrevABC*-transduced cells, which highlights the advantage of multiplexing gRNA to increase gene-editing efficiency.

## Discussion

In the present study, we demonstrated that *tat* and *rev*-targeting CRISPR/Cas9 lentiviral constructs successfully abolished regulatory protein expression and function, and inhibited HIV-1 replication in persistently infected CD4^+^ T-cell lines as well as latently infected T cells. The regulatory proteins Tat and Rev play a crucial role in HIV-1 transcription and latency maintenance, and their genomes are among the most functionally conserved across subtypes, making them a compelling site of attack to suppress HIV-1. Our design selection of highly conserved *tat* and *rev* sequences was based on the HXB2 reference strain, and throughout this study, the constructs were shown to be effective against the HIV-1 NL4-3 strain integrated into 293 T and HeLa cells, the LAV strain inside L-2 cells, and the R7 strain inside J-Lat cells.

Previous studies suggested that CRISPR cleavage inside HIV-1 LTR cut out the whole length of the HIV-1 proviral genome by inducing DSB at both ends^25,43^. However, subsequent studies^44^ showed that sequencing analyses after LTR cleavage failed to reveal this excision and, thus, genome editing inside integral genes of HIV-1 may be more efficient to inhibit replication. The results of multiplexing gRNAs also failed to show any excision between *tat* and *rev*, presumably due to the host DNA repair mechanism after CRISPR-induced mutations. Other studies tested CRISPR attacks at the RRE inside the *env* gene to suppress nuclear export^44^; however, a secondary structure instead of the primary sequence is needed in order to maintain the Rev-RNA interaction, and base variations among RRE elements may only have subtle effects on Rev responsiveness^45^; therefore, targeting *rev* directly may be more efficient to achieve this purpose. One study also found that CRISPR/Cas9 transfection targeting the second exon of *rev*, at a site close to *revA* gRNA tested in this study, exhibited the highest degree of mutation out of *LTR*-, *pol-*, and *rev-*targeting gRNAs^46^.

In the present study, we demonstrated that *revB* and *tatA* gRNAs achieved the strongest HIV-1 suppression in persistently and latently infected T cells, respectively. *tatB* and *revA* were initially predicted to exert the highest replication inhibition due to the perceived importance of the targeted motifs for protein function^47,48^. However, other domains, such as the *tat* N-terminal acidic and *rev* multimerization domains, targeted by *tatA* and *revB* are also regarded as integral motifs^49,50^, and other factors, including CRISPR efficiency and mutation characteristics, may eventually play a larger role in influencing inhibition outcomes. Although sequencing showed an overall high frequency of mutations, it currently remains unclear whether the numbers of unique mutations, types of indels, or rates of frameshift mutations are mainly responsible for the varying degrees of efficiency between different gRNAs. The on-target analysis conducted in the present study was limited to Sanger sequencing, and the next generation sequencing of amplicons may provide a deeper understanding of mutation profiles and their relationship with CRISPR efficiency. However, replication inhibition was the most efficient by combining six *tat-* and *rev-*CRISPR gRNAs in a multiplex gRNA lentiviral cocktail.

Our CRISPR construct also showed efficacy in latently infected J-Lat 10.6 cells, proving that the lentiviral delivery of the CRISPR/Cas9 system successfully reached and cleaved the dormant HIV-1 proviral genome. The inaccessibility of transcriptionally inert HIV-1 DNA due to a modified chromatin structure was previously considered to inhibit gene editing, and a “shock and kill” approach using latency-reversing agents may be necessary^16^. However, the results of the present study suggest that CRISPR has the ability to target HIV-1 DNA without prior reactivation. The proviral genome found in latent infection is mostly defective^51^, whereas latently infected cell populations harboring intact *tat/rev* regions are capable of producing the virus upon the reversal of latency^52^, and targeting these genes to suppress reactivation is of significant value. Replication inhibition for several gRNAs, particularly *rev*-CRISPR, is weaker than that observed in persistently infected L-2 cells, which may prove the difficulty of genomic access to some extent. Another study also described a successful CRISPR attack on unstimulated latently HIV-1-infected J-Lat cells, albeit via nucleotransfection^46^. The capability of lentiviral vectors to transduce non-dividing cells, including resting CD4^+^ T cells, and maintain stable, long-term Cas9 transgene expression supports their potential use in eradicating infected cells constituting the latent reservoir. Cas9 expression was found to vary to some extent (Fig. 2A), but did not appear to correlate with CRISPR efficiency, which may further highlight the importance of the gRNA sequence. It is important to note that clonal cell lines were used in the present study, in which HIV-1 was integrated within specific sites in cellular DNA, and it currently remains unclear whether CRISPR efficiency is affected by differences in integration sites inside host DNA loci. However, our results showed viral suppression in both the latent state, in which HIV-1 DNA is frequently integrated in or near heterochromatin, and the persistent, transcriptionally active state, in which integration in the nuclear periphery is disfavored^13,53^.

The CRISPR recognition mechanism may tolerate certain mismatches to a varying degree and produce off-target mutagenesis, and the selection of a highly specific gRNA design is important for minimizing this effect. In the present study, simple Surveyor nuclease screening was used to show the absence of mutations in two of the most probable off-target sites of each gRNA inside the human genome. However, the Surveyor assay only serves as a preliminary mutation screening and may miss some mismatches in a heteroduplex DNA^54^. Assay results heavily depend on the quality of PCR amplicons and band intensities may underestimate the actual mutation events, as shown in the on-target lanes in Fig. 4. A comprehensive evaluation of off-target mutations may require deep whole-genome sequencing. Insertional mutagenesis potentially caused by a lentivirus may also cause genotoxicity and oncogenesis^55^; however, the cell viability assay performed in this study showed similar growth between WT and lentivirus-transduced cells.

Latent HIV-1 is a static sequence and non-evolving, with the lack of reverse transcription generally indicating that resistance-associated mutations do not occur^56^. However, similar to its capacity to escape from antiretroviral monotherapy and other gene editors, e.g. ZFN^57^, several novel findings have shown that mutations by NHEJ repair or a silent mutation occurring at the PAM recognition site may induce resistance to subsequent CRISPR attacks^58,59^. However, more recent studies discovered that the combination of two gRNAs circumvented this issue because repeated Cas9 cleavage may have led to the saturation of target mutations and prevented viral escape, analogous to the combination ART strategy. Dual gRNA combinations, particularly those targeting conserved and essential viral gene sequences, are able to mutate a large fraction of the provirus and inactivate it, creating major bottlenecks in viral evolution by severely limiting replication-competent virus variants^60^. In the present study, we combined even more gRNAs; six *tat* and *rev* gRNA combinations showed the similar or even stronger inhibition of replication than single gRNA. Viral escape is mainly facilitated by mutations that are non-deleterious to HIV-1 and, thus, the targeting of integral regulatory genes may minimize this effect because *tat/rev* dysfunctions inherently inactivate transcription and are primarily responsible for post-integration latency^61^. However, a viral resistance analysis was not performed in this study and repeated HIV-1 infections to CRISPR-transduced cells may shed further light on the emergence of *tat* and *rev* gRNA-specific escape mutants.

In conclusion, the results of the present study showed that the CRISPR/Cas9 system is robust and efficient for targeting the HIV-1 proviral genome to suppress replication in latency models. CRISPR efficiency mainly depends on how well the gRNA sequence matches the target DNA, and targeting of the highly conserved regulatory genes *tat* and *rev* is beneficial for this effect. Moreover, the importance of these genes to the transcription ability of HIV-1 may lessen the emergence of CRISPR escape mutants. The combination of multiple gRNAs may also maximize efficiency and minimize the risk of resistance. Recent studies may have proven efficacy in cell cultures and *ex vivo*; however, when *in vivo* safety and efficacy profiles have been established, CRISPR-bearing lentiviral vectors may be delivered into HIV-1-infected individuals to clear latent viral reservoirs. Based on the rapid advances being achieved in CRISPR/Cas9 research, a HIV-1 functional cure may soon be within reach.

## Methods

### gRNA designs and plasmids

Target sites from inside the *tat* and *rev* genes of the HIV-1 reference strain HXB2 genome (GenBank accession no. K30455), either the sense or antisense strand, were identified using the CRISPR design tool at http://crispr.mit.edu ^62^. Homology across subtypes was obtained by aligning the designed gRNAs to 97 HIV-1 strains from the Los Alamos National Lab (LANL) HIV Sequence Compendium using the QuickAlign v2 tool^63^, and percentage values signify the number of conserved nucleotides in the 23-bp on-target sequence. Nucleotide variability as measured by the average Shannon entropy score is also shown; a higher score indicates greater variability. Synthetic gRNA oligonucleotides were cloned into pLentiCRISPRv2 (Addgene #52961) at BsmBI restriction sites^64^.

### Lentiviral vector construction and transduction

Lentiviral vectors were produced by co-transfecting 293 T cells with a cocktail of gRNA/Cas9-expressing lentiCRISPRv2, the Gag-Pol packaging plasmid psPAX2 (Addgene #12260), and VSVG expression vector pHIT/G^65^ at a ratio of 3:4:1 using FuGENE HD transfection reagent (Promega).

Transduction of the lentiviral vector into target cells was performed with MOI 10. MOI of 10 was selected because it was previously reported to be most optimal for expressing the transgene into target T cells with minimal cytotoxicity^39^. A preliminary analysis to assess the transducing unit (TU) was performed by transducing a GFP-bearing lentiviral vector in titration into 293 T cells and observing GFP expression under a fluorescent microscope in order to obtain TU/mL, before using it to calculate MOI. LentiCRISPRv2 without gRNA was used as an empty vector for mock transduction. Culture medium containing the virus was maintained for 5 days; second transduction was performed 7 days later, and the virus was also maintained for 5 days before proceeding with the analysis. Antibiotic selection was not performed after each transduction.

### Multiplex gRNA transduction

Multiple gRNA transductions were performed by the co-infection of three *tat* and *rev* gRNA-bearing lentiviral vectors (*tatABC* and *revABC*) or six gRNAs (*tatrevABC*) in a lentiviral cocktail. MOI was the same in all multiplex gRNA groups as those in the single gRNA treatment groups. While MOI of 10 was used for single gRNA transduction, a 1/3 titer of each gRNA/CRISPR vector for *tatABC* and *revABC*, i.e. MOI 3.33 for each lentivirus, and a 1/6 titer of each vector for *tatrevABC*, i.e. MOI 1.67, for each lentivirus were used.

### Cell cultures and development of stable Tat- and Rev-expressing cells

HEK 293 T and HeLa cells were grown in Dulbecco’s modified Eagle’s medium (DMEM; Nacalai Tesque) supplemented with 10% fetal bovine serum (FBS; BioWest). L-2 cells were grown in RPMI 1640 medium (Nacalai Tesque) with 10% FBS. J-Lat 10.6 cells were obtained from the NIH AIDS Research and Reference Reagent Program (ARRRP), Division of AIDS, NIAID, and were grown in RPMI 1640 medium with 10% FBS, 100 U/mL penicillin G, and 100 μg/mL streptomycin.

The *tat* and *rev* genomes were amplified from pNL4-3^66^ and FLAG-tagged at the N- and C-terminal ends, respectively, before being cloned into the pLenti CMV Puro DEST backbone (Addgene #17452)^67^. Co-transfection into packaging 293 T cells was performed with psPAX2 and pHIT/G to produce VSVG-pseudotyped lentiviral vectors. Transduction into target 293 T and HeLa cells was performed with MOI of 1 and selected with puromycin. Limiting dilutions were performed and the strongest FLAG-expressing cells were selected by immunoblot assays.

### Antibodies for Western blotting

Protein samples were separated by SDS-polyacrylamide gel electrophoresis (SDS-PAGE) and transferred to a polyvinylidene fluoride (PVDF) membrane, blocked with 5% non-fat milk in PBS, and immunostained with the primary antibody as follows: an anti-FLAG M2 monoclonal antibody (Sigma Aldrich) for FLAG expression, CRISPR/Cas9 polyclonal antibody (Epigentek) for Cas9, anti-HIV-1_SF2_ gp120^68^ for envelope gp120, and anti-beta-actin polyclonal antibody (BioVision) for beta-actin. Anti-HIV-1_SF2_ gp120 was obtained from NIH ARRRP. Primary antibody binding was followed with an incubation with peroxidase-labeled secondary antibodies and an immunocomplex was visualized with Pierce western blotting substrate (Thermo Scientific) using the OptimaShot CL-420α chemiluminescence imaging system (Wako). Blot images were cropped using Photoshop CS6 and any contrast adjustment was applied equally across the entire image, including control lanes.

### Tat and Rev functional analysis

A Tat functional analysis was performed by transfecting target cells with pLTR-Luc, and three days after transfection, cells were lysed to measure luciferase activity. The plasmid pLTR-Luc contains HIV-1 3′LTR upstream of the *luc* gene and transactivation by Tat will drive luciferase expression^69^. Transfections of the mammalian expression vector pRc/RSV (RDB No 580, Riken Gene Bank) and the Tat expression vector pRSV-Tat^70^ into 293 T or HeLa cells were used as negative and positive controls, respectively.

A Rev functional analysis was performed by transfecting target cells with the gp140-expressing plasmid, and three days after transfection, the cell lysate was immunostained with an anti-gp120 antibody. The plasmid gp140 was constructed from wild-type gp160, which was terminated at the N terminus to the membrane-spanning region, had a serine substitution at the gp120-gp41 junction, and attached with the T4 bacteriophage fibritin at the C terminus in order to stabilize uncleaved gp140 trimers^71^. Transfections of pcDNA3.1/Zeo(+) (V86020, Invitrogen) and the Rev expression plasmid pCA-Rev^72^ into 293 T cells were used as negative and positive controls, respectively.

### Reactivation for latent infection

Cytokine reactivation for latently infected J-Lat cells was performed by adding 20 ng/mL of TNFα into cells with fresh medium, which was conducted five days after the gRNA/CRISPR treatment. Twenty-four hours after reactivation, the supernatant was cleared from cell debris by centrifugation at 3000 RPM for 5 minutes, and p24 inside the supernatant was measured as described below.

### p24 ELISA

HIV-1 p24 was quantified using anti-p24 ELISA (ZeptoMetrix RETROtek HIV-1 p24 antigen ELISA, Fisher Scientific) according to the manufacturer’s protocol. The timing of the p24 measurement was 3 days after the fresh medium change for L-2 cells and 24 hours after cytokine reactivation for J-Lat cells.

### On- and off-target analyses

Genomic DNA was extracted from CRISPR-transduced and control cells 7 days after transduction using the QIAamp DNA Blood Minikit (Qiagen) before being used as templates for on- and off-target PCR. On-target amplicons were sequenced (Macrogen Inc.) and analyzed using GENETYX software ver. 10 (GENETYX Corp.).

Potential off-target sites inside the coding region of the human genome were identified using the aforementioned CRISPR design tool^62^. Two sites with the highest off-target probability from each gRNA were selected. PCR primers were designed to amplify 400–800-bp sequences from the human genome that contained each off-target site (Supplementary Table 1). Amplicons were hybridized before being digested with nuclease from the Surveyor mutation detection kit (IDT). Products were then analyzed on a 2% agarose gel, stained with GelRed (Biotium), and photographed.

### Statistical analysis

All experiments in the present study were conducted with at least 3 replicates and data were shown as means ± standard deviations (SD). A one-way analysis of variance (ANOVA) was performed for samples from more than 2 groups, and pairwise comparisons using an independent-sample *t*-test were conducted if the ANOVA test gave a *p* value < 0.05. All analyses were performed with SPSS 23.0 (SPSS, Inc.).

### Data availability

All necessary data generated or analyzed during the present study are included in this published article and its Supplementary Information files.

## Electronic supplementary material

Supplementary Information
