# Supplementary material for: CRISPR/Cas9 system targeting regulatory genes of HIV-1 inhibits viral replication in infected T-cell cultures
Source: Sci Rep. 2018 May 17;8:7784. doi: 10.1038/s41598-018-26190-1 (PMC5958087; doi:10.1038/s41598-018-26190-1)

**CRISPR/Cas9 system targeting regulatory genes of HIV-1 inhibits viral replication in infected T-cell cultures**

**Supplementary Information**

Youdiil Ophinni,^1^ Mari Inoue,^2^ Tomohiro Kotaki^2^, Masanori Kameoka^1,2,a^

^1^*Center for Infectious Diseases, Kobe University Graduate School of Medicine, Hyogo 650-0017, Japan*

^2^*Department of International Health, Kobe University Graduate School of Health Sciences, Hyogo 654-0142, Japan*

^a^Corresponding author: Masanori Kameoka

Department of International Health, Kobe University Graduate School of Health Sciences,

7-10-2 Tomogaoka, Suma-ku, Kobe, Hyogo 654-0142, Japan

Tel./Fax: +81-78-796-4594
E-mail: [mkameoka@port.kobe-u-ac.jp](mailto:mkameoka@port.kobe-u-ac.jp)

**
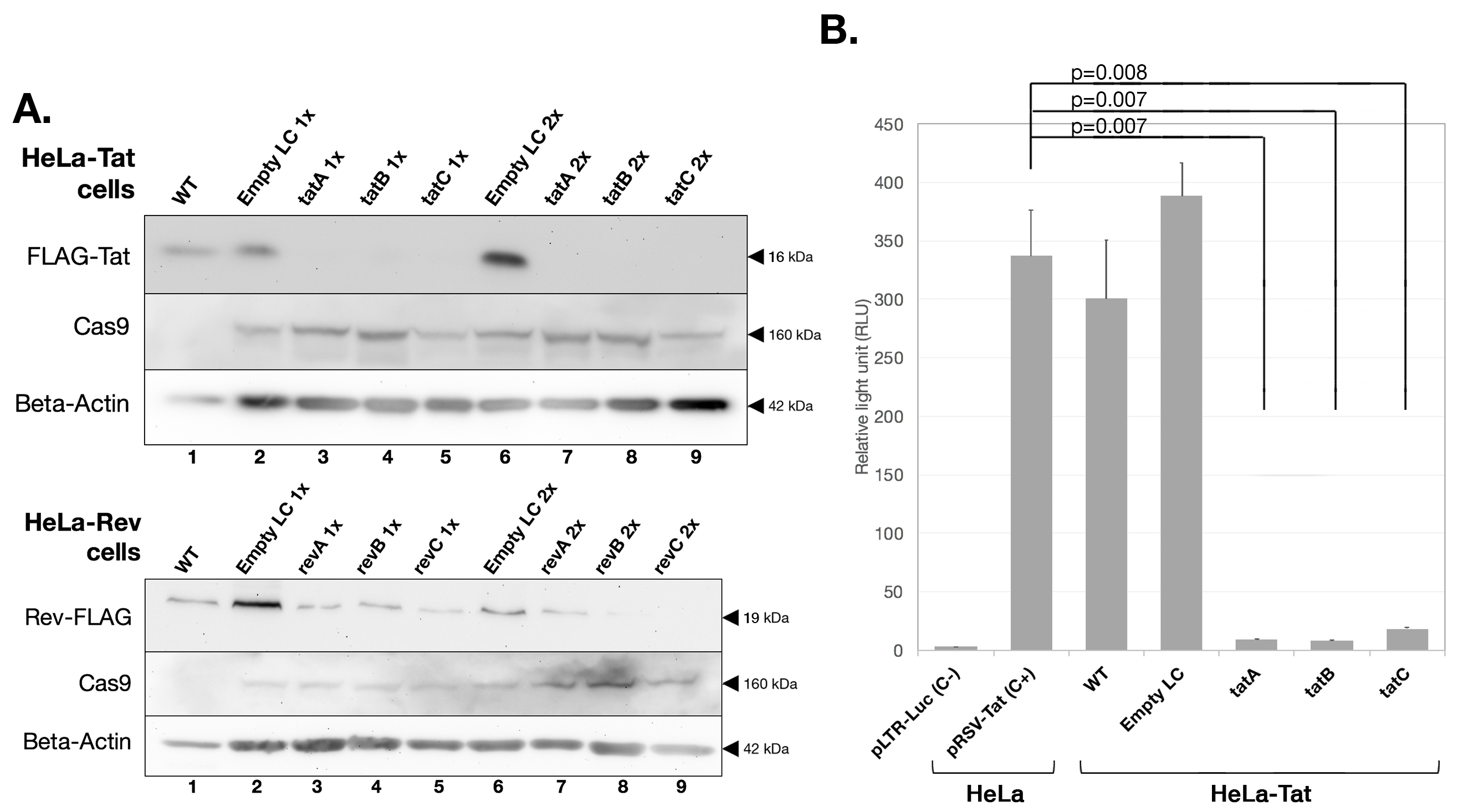
**

**Fig. S1.** CRISPR/Cas9 abolished Tat and Rev protein expression in stable Tat- and Rev-expressing transformant 293T cells. **(A)** Western blot analysis for FLAG-bound Tat and Rev protein expression. Transformant HeLa cells were transduced with a CRISPR/Cas9-bearing lentiviral vector for 3 days before being lysed and immunostained with an anti-FLAG antibody. The transduction of lentiCRISPR without gRNA (empty LC) and with *tat*/*rev*-targeting gRNAs was performed once or twice, and protein expression was compared with non-transduced cells (WT). The Cas9 protein was detected by the anti-Cas9 antibody and expression was shown for each cell group. Beta-actin expression was used as a loading control. The original unedited blots are shown below as the supplementary dataset and images were not joined from different parts of gel. **(B)** Tat functional analysis in HeLa-Tat cells. WT and CRISPR-transduced cells were transfected with pLTR-Luc for 3 days and the cell lysate was obtained to measure luciferase activity. The transfection of pRc/RSV and the Tat expression vector pRSV-Tat into original HeLa cells were used as a negative and positive control, respectively. HIV-1 LTR-driven expression was suppressed even further than that by 293-Tat cells: 97%, 97%, and 94% (P=0.007, P=0.007, and P=0.008) for the *tatA*, *tatB*, and *tatC* constructs, respectively.

**Table S1. List of off-target sites for each gRNA tested in this study.**

| **Guide RNA and respective off-target sites** | **Sequence** | **Off-target score^+^** | **Number of nucleotide mismatches and respective locations** | **Locus in the human genome** |
| --- | --- | --- | --- | --- |
| ***tatA***  ***tatA* off-target 1**  ***tatA* off-target 2** | TAGATCCTAGACTAGAGCCCTGG  TAGAACCCAAATTAGAGCCCCAG  CAGATCCAGGACTAAAGCCCGGG | 0.6  0.6 | 4 (5,8,10,12)  4 (1,8,9,15) | Chr1(q21,3) -155,659,924  Chr5(p11,2) -195,403 |
| ***tatB***  ***tatB* off-target 1**  ***tatB* off-target 2** | CCTTAGGCATCTCCTATGGCAGG  ATTTGGGCAACTCCTATGGCGAG  GGTTCGGCTTCTCCTATGGCAGG | 1.3  0.9 | 4 (1,2,5,10)  4 (1,2,5,9) | Chr11(q21,3) +45,869,358  Chr1(p11,2) +150,283,517 |
| ***tatC***  ***tatC* off-target 1**  ***tatC* off-target 2** | GCAGTTTTAGGCTGACTTCCTGG  GCTGTCTTAGGCTGACTTCTCAG  GCAGTCGAAGGCTGACTTCTAGG | 0.9  0.3 | 3 (3,6,20)  4 (6,7,8,20) | Chr12(q21,3) +57,070,576  Chr17(p11,2) -74,314,212 |
| ***revA***  ***revA* off-target 1**  ***revA* off-target 2** | TCTATTCCTTCGGGCCTGTCGGG  TCTAGTCCTTCCTGCCTGCCTAG  TCTTTTCCTTTGGGCCAGCCTGG | 0.1  0.1 | 4 (5,12,13,18)  4 (4,11,17,19) | Chr17(q21,3) -42,759,668  Chr15(p11,2) -65,391,793 |
| ***revB***  ***revB* off-target 1**  ***revB* off-target 2** | CACTTATCTGGGACGATCTGCGG  CCCTTGTATGAGACGATCTGAGG  AACTTGTCTGGGCCCATCTGGGG | 0.5  0.1 | 4 (2,6,8,11)  4 (1,6,13,15) | Chr1(q21,3) -152,911,577  Chr11(p11,2) -45,931,291 |
| ***revC***  ***revC* off-target 1**  ***revC* off-target 2** | AGAGTAAGTCTCTCAAGCGGTGG  AGTGTCAGTCACTCAAGCTGGGG  GGAGTCAGTCTCTCCAGAGGCAG | 0.2  0.1 | 4 (3,6,11,19)  4 (1,6,15,18) | Chr5(q21,3) -139,855,650  Chr7(p11,2) +156,681,929 |

^+^Off-target score represents the probability of off-target mutagenesis, higher score means higher probability. Scores were calculated using the algorithms available in http://crispr.mit.edu.

**Original Western Blots**

**Original Western Blots image of Fig. 2A**

Original unedited Western blot images of Fig. 2A. Framed area indicated the cropped image as seen in Figure 2. No figures were cropped and joined together from different images. Blotted PVDF membranes were incubated with anti-FLAG M2 antibody and anti-Cas9, with anti-beta-actin as loading control. Contrast adjustment using the auto-contrast tool in Photoshop CS6 was applied equally across the entire image, including control lanes.


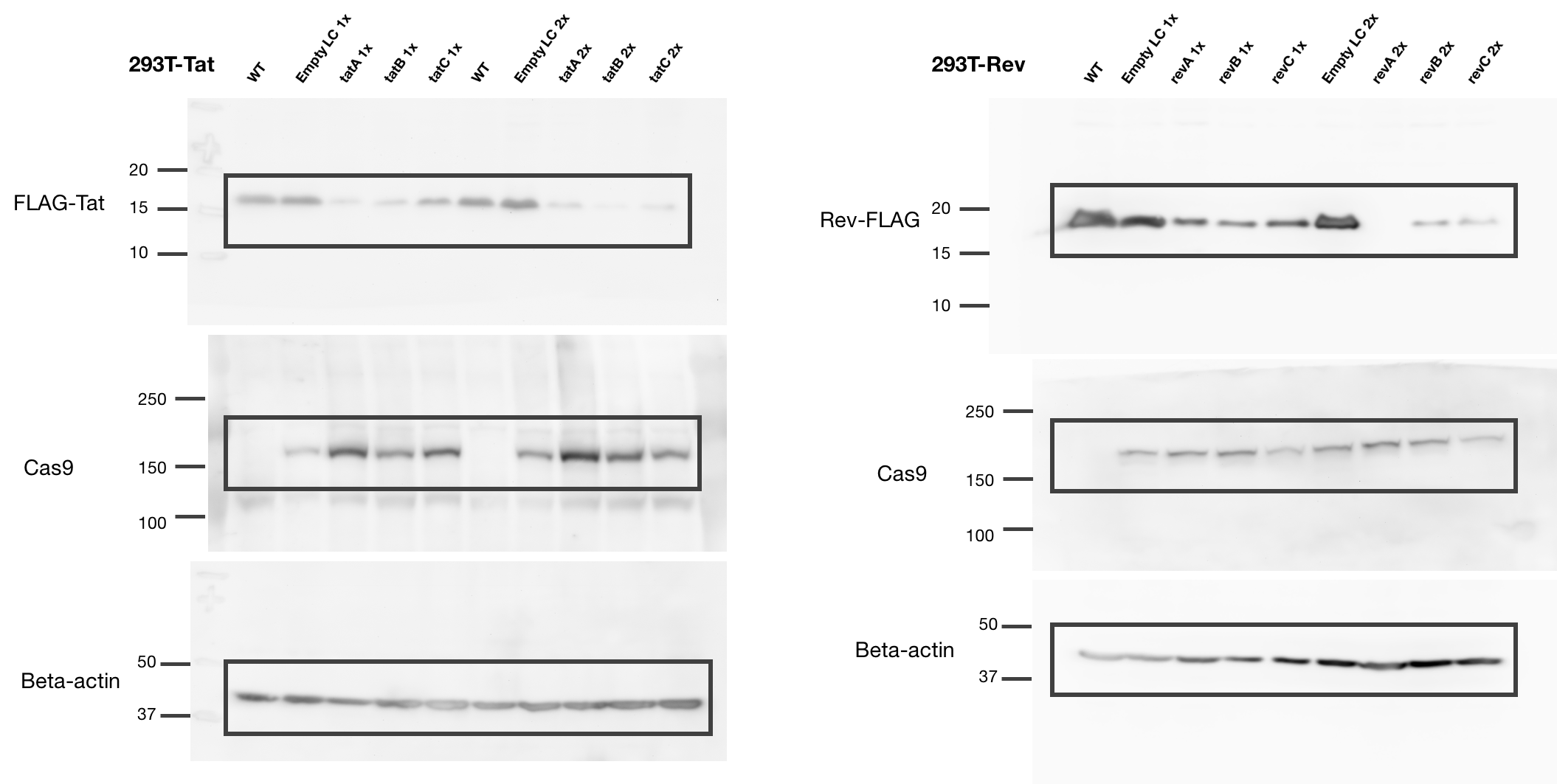


**Original Western Blots image of Fig. 2C**

Original unedited Western blot images of Fig. 2C. Framed area indicated the cropped image as seen in Figure 2. Blotted PVDF membranes were incubated with anti-gp120, and anti-beta-actin as loading control. Contrast adjustment using the auto-contrast tool in Photoshop CS6 was applied equally across the entire image, including control lanes.


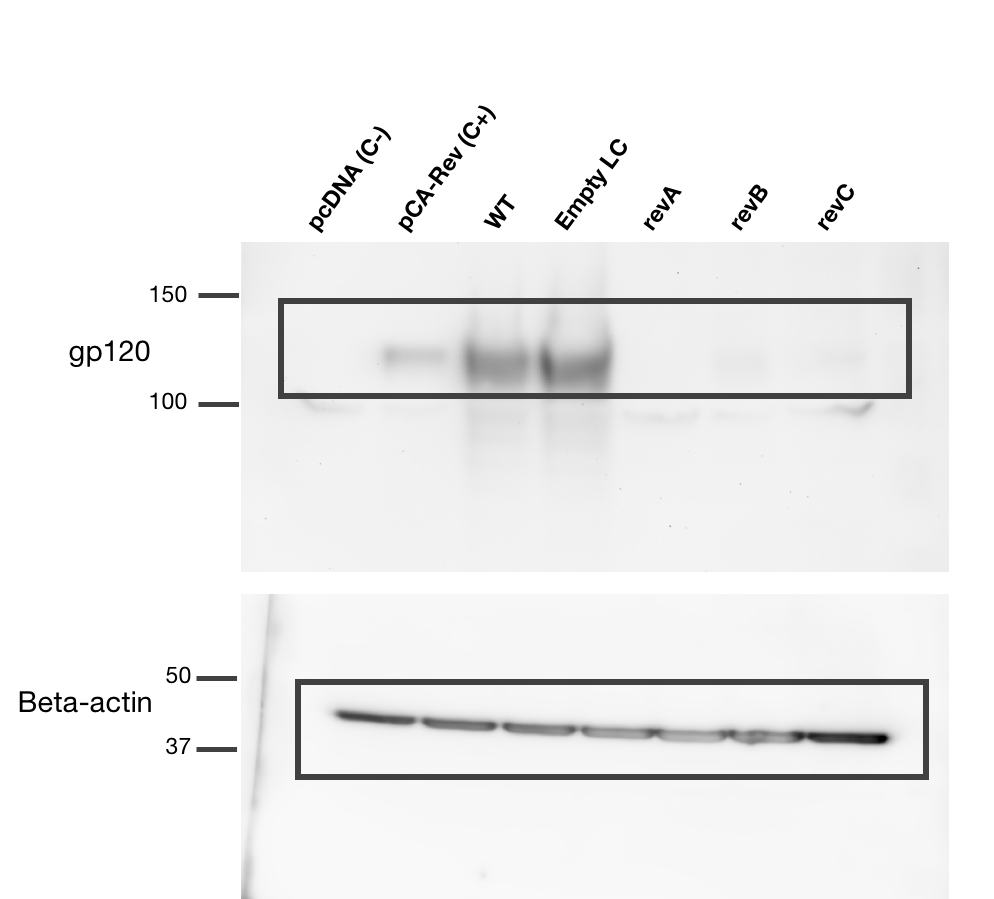

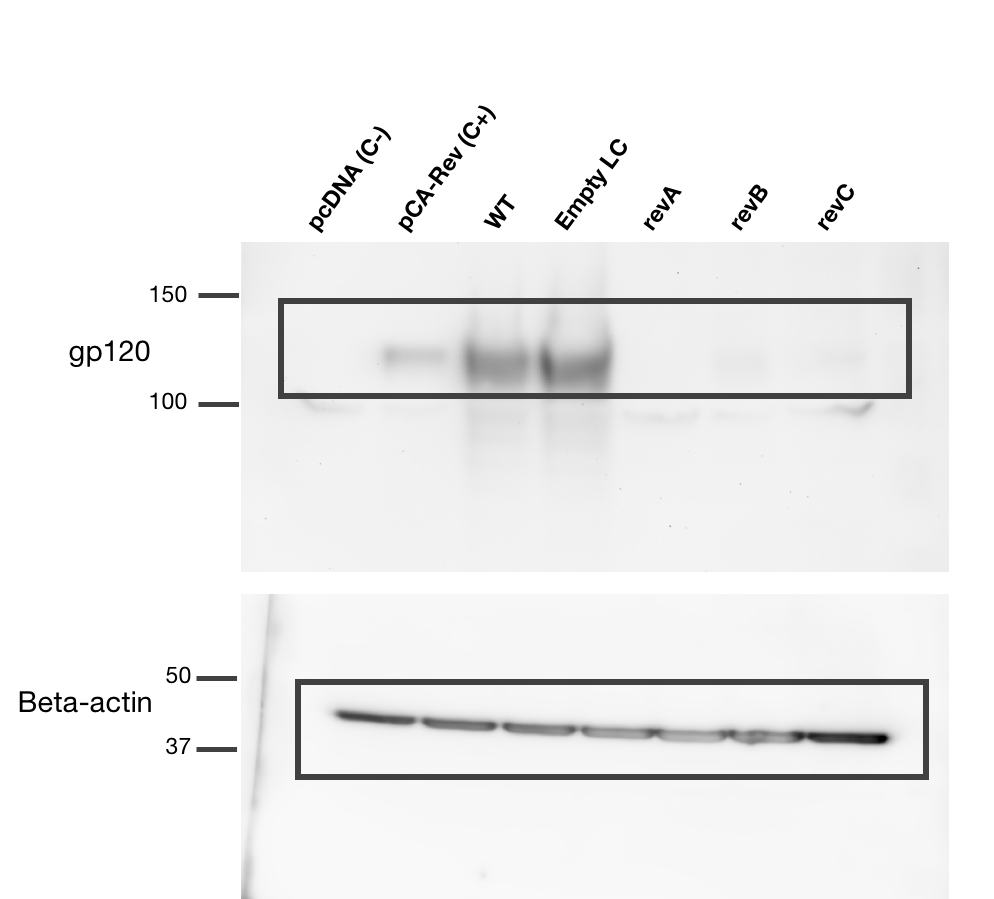


**Original Western Blots image of Fig. S1**

Original unedited Western blot images of Fig. S1. Framed area indicated the cropped image as seen in Figure 2. Blotted PVDF membranes were incubated with anti-FLAG M2 antibody and anti-Cas9, with anti-beta-actin as loading control. Contrast adjustment using the auto-contrast tool in Photoshop CS6 was applied equally across the entire image, including control lanes.


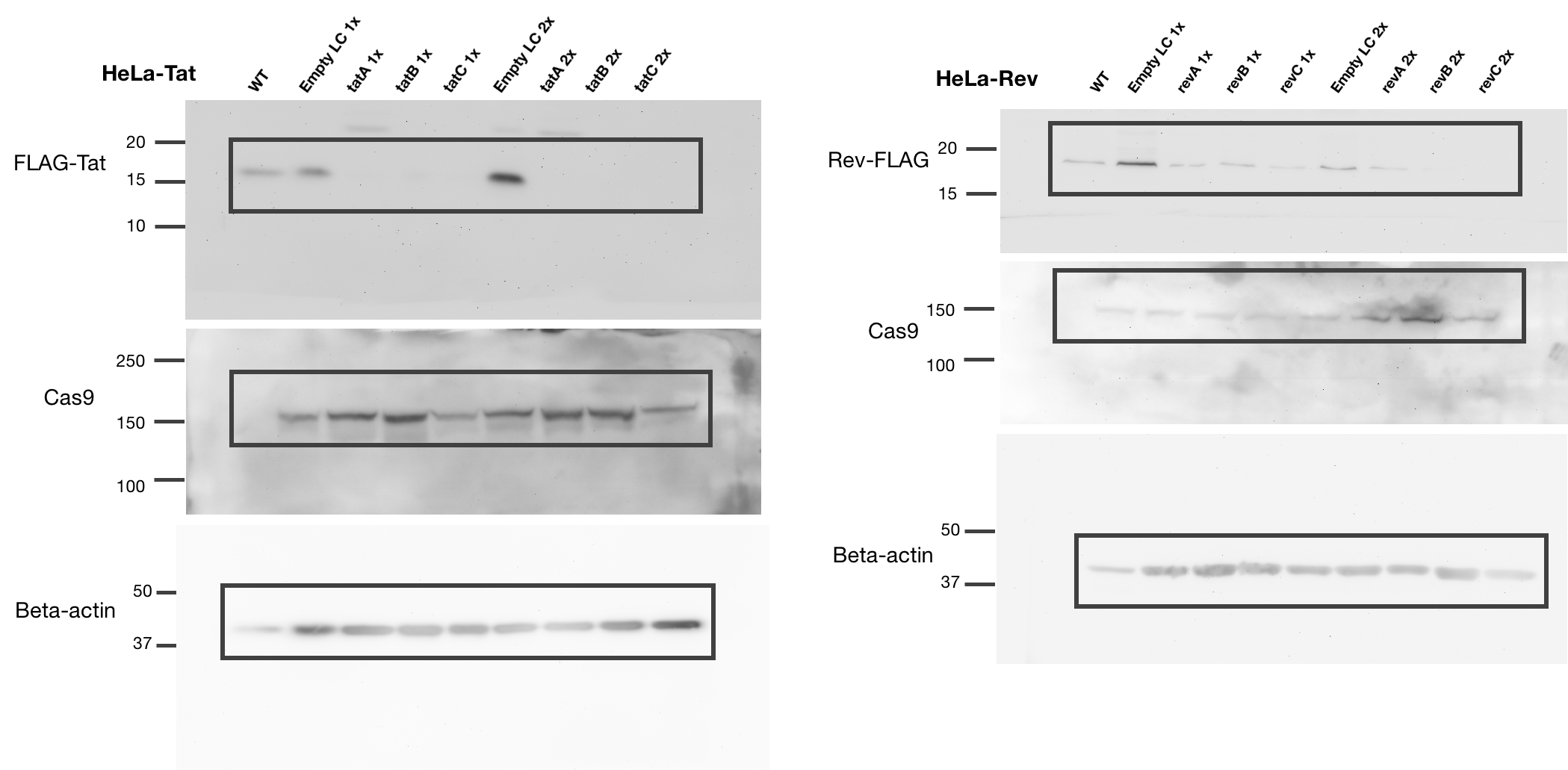

Supplement: Supplementary file 1 — Supplementary Information [file 41598_2018_26190_MOESM1_ESM.doc]
